# Knowledge on leprosy and its management among primary healthcare providers in two districts of Bangladesh

**DOI:** 10.1186/s12913-019-4525-z

**Published:** 2019-11-04

**Authors:** Humayun Kabir, Shahed Hossain

**Affiliations:** 1 Health Systems and Population Studies Division, International Centre for Diarrhoeal Disease Research, icddr,b, 68 Shaheed Tajuddin Ahmed Sarani, Mohakhali, Dhaka 1212 Bangladesh; 2 James P. Grant School of Public Health, BRAC University, icddr,b, Dhaka, Bangladesh

**Keywords:** Leprosy, Bangladesh, Neglected tropical diseases, Primary healthcare providers, Technical assistants, Knowledge

## Abstract

**Background:**

In 2013, Lepra Bangladesh (a non-government organization) and the National Leprosy Programme of the Directorate General of Health Services under the Ministry of Health and Family Welfare, Bangladesh implemented a 3 years project entitled “2015 and Beyond: Poverty Reduction through Strengthened Health Systems”. The aims of this Health System Strengthening (HSS) project were to improve quality of leprosy services through service delivery, capacity development, curriculum development, improved collaboration, coordination, operational research and knowledge sharing to identify and treat leprosy in order to contribute to strengthen existing health systems. We evaluated the changes in knowledge of primary and community level healthcare providers about cardinal signs, course of leprosy treatment, and drug use for paucibacillary (PB) and multibacillary (MB) leprosy cases.

**Methods:**

We conducted two surveys using purposive sampling technique in two pilot districts: Bogra and Moulvibazar. The first survey was conducted before implementing the HSS project from March to June 2014 among 98 providers. The end-line survey was conducted in November 2015 and included 49 providers. The interview was conducted using the same pre-tested structured questionnaire. Descriptive statistics followed by further analysis was done including proportions, 90% confidence intervals, and *p* values were calculated for the selected variables.

**Results:**

The primary and community level healthcare providers demonstrated significant increases in knowledge on one cardinal sign (definite loss of sensation in a pale -hypopigmented- or reddish skin patch), doses and courses for the adult PB and MB cases and duration of Multi-Drug Therapy (MDT) course at the end line compared to the beginning of the project. All the providers except TB and Leprosy Control Assistants demonstrated statistically significant decreases in knowledge at the end-line compared to the baseline about supportive counseling.

**Conclusions:**

HSS activities including training and capacity building of the providers recorded significant increase of knowledge on types of leprosy, one cardinal sign, courses of MDT and drug use for the adult PB and MB cases and use MDT for leprosy treatment among the service providers at the end-line. Any health systems strengthening project should incorporate a capacity building approach within the programme all through.

## Background

Leprosy is a chronic infectious disease, caused by the bacillus *Mycobacterium leprae*. Leprosy transmitted through droplets from the nose and mouth of an untreated person affected by the disease to her close contacts. Physical and sensory disability, including damage to fingers and toes, contractures, inability to close the eyelids and blindness can occur due to delay in treatment of the disease. In 2016, globally a total of 214,783 new cases were reported; over half of them were from India (135,485) alone. However, 16 other countries with pockets of high endemicity were reported including Bangladesh. A decrease in new case detection although has been reported in the South-East Asia Regions during 2002 and 2005, disabilities (grade-2) among the new cases were reported as 1.7 per million population in 2016. The decline of new case detection was probably associated with the integration of leprosy diagnosis and treatment into general healthcare services in global priority countries, combined with the World Health Organization (WHO) target of elimination of leprosy as a public health problem (i.e. a prevalence of < 1 in 10,000 to be achieved by 2000). On the other hand, actual numbers of people affected by the disease is likely to be far higher than statistics show as there still prevails lack of awareness about the disease, lack of skills of general health staff in leprosy diagnosis, inadequate active case findings, lack of inclusion of cases from private sector and presence of high stigma in the community [1].

Bangladesh achieved the WHO leprosy elimination goal in 1998 nationally, with the exception of only one district registered a prevalence of > 1/ 10,000 population in 2016. The estimated prevalence was 0.20 per 10,000 population and the new case detection rate was 2.0 per 100,000 population at the end of 2013 which remained similar to the end of 2016 (Hossain et al: Leprosy in Bangladesh 2014: a situation analysis, unpublished; Bangladesh: Directorate General of Health Service. National Leprosy Elimination Programme.NLEP annual disease profile report 2016, unpublished). However, it is assumed that there might be more hidden or undetected cases than reported.

In Bangladesh the National Leprosy Programme (NLP) falls under the Directorate General of Health Services (DGHS), executes elimination activities according to the WHO guidelines and administering the Multi-Drug Treatment (MDT) since 1993. Leprosy services were integrated with the general health services at all levels under the 5 years (1998–2003) Health Population and Sector Programme (HPSP). Despite the availability of vertical staff engaged in the control programme, the NLP has gradually slipped from the public health agenda, subsequently deteriorating leprosy services. This deterioration of emphasis and priority is most markedly demonstrated in the loss of skilled personnel at field level [2]. Some of the NLP partner Non Government Organizations (NGOs) continue to provide services in most of the endemic districts of the country and, thus NGOs detect nearly 80% of the cases after the integration took place; however, these NLP partner NGOs faced enormous financial and other resource constraints, which deemed to reduce the quality of services available to the population (Bangladesh: Directorate General of Health Service. National Leprosy Elimination Programme.NLEP annual disease profile report 2017, unpublished).

The WHO mentioned in the enhanced global strategy for Leprosy (2011–2016) that there is loss of clinical skills in recognizing and managing leprosy and its complications, lack of interest by the young doctors to specializing in leprosy, lack of research, less political commitment as major challenges to reduce the leprosy burden. Investment in the leprosy services are now reducing among many governments, resulting in declining professional expertise and knowledge of the disease [3].

In 2013, Lepra Bangladesh, an NGO partner of the NLP implemented a 3 years project (2013–2015) “2015 and Beyond: Poverty Reduction through Strengthened Health Systems” (hence forth the HSS Project). The aims of the project were to improve the quality of leprosy services through the integration of service delivery at all the MDT centres through capacity development, curriculum development, improved collaboration and coordination, operational research and knowledge sharing activities to identify and treat leprosy in order to strengthen health systems for leprosy. This 3 years project was implemented in 12 districts of Bangladesh jointly by the Ministry of Health and Family Welfare (MoHFW), the NLP, and overlooked by the National Leprosy and TB Coordinating Committee (NLTCC), NGOs, icddr,b and the WHO. Under this project six NGOs were assigned on a comprehensive, functional and fully integrated response to leprosy management in ten districts. At the initial stage of the HSS project, icddr,b conducted a base-line survey on situation analysis of leprosy in Bangladesh and a comprehensive disease mapping. The base-line findings provided a comprehensive epidemiological and geographical distribution of leprosy in Bangladesh and also provided information on the social aspect of the disease, like care seeking, knowledge and associated stigma in the community, among the persons affected by leprosy (PABL), the knowledge and practices by the service providers and policy makers. The findings helped identifying many gaps and challenges in leprosy care in Bangladesh (Hossain et al: Leprosy in Bangladesh 2014: a situation analysis, unpublished). Based on these findings, icddr,b developed strategies to facilitate strengthening of the integration of leprosy services (a pilot model) into general health systems. An operational research was undertaken by icddr,b to capture the impacts of piloting the integration of leprosy services into general health care during the month of July 2014 to October 2015 i.e. 15 months in two pilot districts: Bogra under Rajshahi division and Moulvibazar under Sylhet division. This paper captured parts of the findings from the baseline and the end-line surveys conducted in these two pilot districts. The specific objective of the present study was to assess the changes in knowledge on leprosy and its management among primary and community level healthcare providers in two pilot districts of Bangladesh at the end of the project period.

## Methods

### Study design and sites

A cross-sectional and pre-post study design was adopted. The study sites were northern endemic district of Bogra under Rajshahi division and eastern district Moulvibazar under Sylhet division of Bangladesh. Seven sub-districts were purposively selected from 18 sub-districts of Bogra and Moulvibazar. In each primary healthcare service centre i.e. Sub-district Health Complex, one TB and Leprosy Control Assistant (TLCA), is assigned as a frontline service provider to detect leprosy cases and provide treatment, maintain records and reports, procure and preserve medicine and other logistics and provide health education. One Medical Officer-Disease Control i.e. Physician is posted in each Sub-district Health Complex for complication management, supervision and monitoring of the leprosy services. Both physician and TLCA were chosen from selected each sub-districts for this study. However a very few villages (community) were purposively selected from seven sub-districts for conducting interview with the selected field workers and their supervisors.

### Study population and duration

Two surveys using purposive sampling technique were conducted to assess the knowledge and skill of the providers who engaged in leprosy services at primary (Sub-district) and community level in two pilot districts. The baseline survey was conducted from March to June 2014 before the inception of the HSS Project. After the baseline survey, under HSS project all the frontline service providers (TLCAs and physicians) received 1 day orientation and 3 days training on aim and objectives of the HSS project, basic facts of leprosy, nervous system and its functions, clinical features of leprosy and diagnosis, classification of leprosy and its management, treatment course, care of hands, feet and eyes, practical demonstration of peripheral nerve examination, referral slip, recording and reporting systems. On the other hand, field workers and their supervisors received only 1 day orientation on aim and objectives of the HSS project, basic concepts on leprosy, referral slip and referral linkage.

End-line survey was conducted in these same pilot districts in November 2015 at the end of the HSS project. The study population was selected purposively from the primary healthcare providers i.e. Physician and TLCA including field staff and their supervisors from seven sub-districts out of 18. Data was collected from three groups of service providers i.e. Physician, TLCA and Others (“Others” mean field workers and their supervisors) from selected sub-districts. More than one-third physicians and TLCAs participated from Bogra and Moulvibazar districts in both the surveys. Some of the field workers and their supervisors were selected from these seven sub-districts. The baseline survey was conducted among 98 service providers i.e. 12 physicians, 6 TLCAs and 80 fieldworkers and their supervisors and at the end of the project 49 service providers i.e. 7 physicians, 7 TLCAs and 35 fieldworkers and their supervisors were included in the survey. Only a few numbers of service providers were included in both the surveys. The sample sizes of the primary healthcare providers were almost same among the baseline (18) and the end-line (14). However, the sample sizes of the field workers and their supervisors were smaller in the end-line (35) than the baseline (80) due to constraint of budget and time.

### Data collection

The interviews were conducted using the same pre tested structured questionnaire in both rounds of the survey. “*Service Provides Survey Questionnaire”*: The questionnaire included knowledge on the diagnosis of leprosy, treatment course, follow-up of the diagnosed cases, leprosy complications, management of complications, referral, and training received on leprosy and their participation in outreach activities (Additional file 1). The interviews were conducted by the trained field research officers (FROs) for this purpose.

### Data analysis

Descriptive analysis was conducted to estimate distributions of relevant characteristics of the service providers. A bivariate analysis was done to compare the baseline and the end-line findings of the HSS project with respect to knowledge on types of leprosy, one cardinal sign, types of leprosy services, course of leprosy treatment, and drug use for PB and MB, and leprosy services. Proportions, 90% confidence intervals (CIs), Chi square tests and *p* values were calculated for selected variables for comparisons between the both surveys. SPSS version 10.0 (IBM Corp, Armonk, NY) was used for all statistical analysis.

## Results

### Background of the survey respondents

Socio-demographic characteristics of the providers are given in Table 1. Overall, most of the TLCAs were above 35 years of age while most of the physicians and other service providers were between 26 and 35 years. At least one-third of all categories of the service providers were female both in baseline and end-line except TLCAs, who were exclusively male at the end-line. Education level of physician was bachelor degree and above. However, education level of the TLCAs and other service providers varied between secondary to bachelor degrees and above.

**Table 1:** Basic characteristics of service providers

Socio-demographic characteristics	Physician		TLCA		Others*	
	Baseline (*n* = 12)%	End-line (*n* = 7)%	Baseline (*n* = 6)%	End-line (*n* = 7)%	Baseline (*n* = 80)%	End-line (*n* = 35)%
Age (Yrs)						
≤ 25	0%	14%	17%	0%	11%	23%
26–35	83%	43%	17%	0%	41%	46%
> 35	17%	43%	66%	100%	48%	31%
Mean age	32	32	34	38	33	29
Sex						
Male	67%	57%	67%	100%	65%	69%
Female	33%	43%	33%	0%	35%	31%
Education						
Secondary	0%	0%	50%	0%	68%	6%
Higher secondary	0%	0%	17%	14%	31%	43%
Bachelor and above	100%	100%	33%	86%	1%	51%

Others* mean field workers and their supervisors

### Knowledge on leprosy among service providers

TLCA’s training was universal as reflected in the baseline and the end-line (Table 2). A significantly higher proportion of other service providers (field workers and their supervisors) received training on leprosy during Health System Strengthening (HSS) Project compared to the baseline (50% vs. 97%; *p* < 0.001). Similarly, more than double of the physicians received the training on leprosy during HSS Project compared to the baseline (25% vs. 57%). All TLCAs had comprehensive knowledge on the types of leprosy as shown in both the surveys. All the physicians could correctly classified leprosy into PB and MB sub-types at the end-line compared to the baseline (50% vs. 100%; *p* < 0.05). Also other service providers demonstrated significantly increased in knowledge on the types of leprosy at the end-line compared to the baseline (11% vs. 89%; *p* < 0.001).

**Table 2:** Comparison of leprosy training and Knowledge about types of leprosy among service providers

	Physician			TLCA			Others*		
Training	Baseline (*n* = 12) % (90% CI)	End-line (*n* = 7) % (90% CI)	*P*-value	Baseline (*n* = 6) %	End-line (*n* = 7) %	*P*-value	Baseline (*n* = 80) % (90% CI)	End-line (*n* = 35) % (90% CI)	*P*-value
Training Received on leprosy	25%	57%	NS	100%	100%	NS	50%	97%	***
	(5.0–46.6)	(26.3–87.9)					(40.8–59.2)	(92.4–101.8)	
Knowledge about types of Leprosy									
Paucibacillary (PB) + Multibacillary (MB)	50%	100%	*	100%	100%	NS	11%	89%	***
	(26.0–73.7)						(5.5–17.1)	(79.8–97.4)	

*NS* Not significant

*p < 0.05; **p < 0.01; ***p < 0.001

Others* mean field workers and their supervisors

### Knowledge about one cardinal sign and duration of MDT course for both types of leprosy

Table 3 shows that TLCAs had inadequate knowledge on one cardinal sign i.e. definite loss of sensation in a pale (hypopigmented) or reddish skin patch in the baseline of the project which subsequently increased significantly at the end-line (33% vs. 86%; *p* < 0.05). A higher proportion of the physicians had increased knowledge on one cardinal sign (67% vs. 71%) and other service providers had also increased knowledge significantly on one cardinal sign at the end-line compared to the baseline (72% vs. 89%; *p* < 0.05) respectively.

**Table 3:** Knowledge about one cardinal sign and duration of MDT course for PB case & MB case

	Physician			TLCA			Others*		
Knowledge about one cardinal sign	Baseline (*n* = 12) % (90% CI)	End-line (*n* = 7) % (90% CI)	*P*-value	Baseline (*n* = 6) % (90% CI)	End-line (*n* = 7) % (90% CI)	*P*-value	Baseline (*n* = 80) % (90% CI)	End-line (*n* = 35) % (90% CI)	*P*-value
Definite loss of sensation in a pale (hypopigmented) or reddish skin patch	67% (35.0–98.4)	71% (43.3–99.5)	NS	33% (10.9–55.7)	86% (63.9–107.3)	*	72% (63.5–0.1)	89% (79.8–97.4)	*
Knowledge about duration of MDT course for two types of leprosy									
6 months for PB	33% (10.9–55.7)	100%	**	100%	100%	NS	18% (10.5–24.5)	71% (58.8–84.0)	***
12 months for MB	42% (18.3–65.1)	100%	**	100%	100%	NS	13% (6.4–18.6)	57% (43.3–70.9)	***
Knowledge about Multi Drug Therapy (MDT)	58% (34.9–81.7)	100%	*	100%	100%	NS	14% (7.5–20.1)	94% (87.9–100.7)	***

*NS* Not significant

*p < 0.05; **p < 0.01; ***p < 0.001

Others* mean field workers and their supervisors

All TLCAs reported about the use “MDT” for leprosy treatment both in baseline and end-line. A significant proportion of physicians reported about the use “MDT” for leprosy treatment at the end-line compared to the baseline (58% vs. 100%; *p* < 0.05). Other service providers demonstrated significant increases in knowledge in the use “MDT” for leprosy treatment at the end-line compared to the baseline (14% vs. 94%; *p* < 0.001).

All TLCAs had adequate knowledge about the duration of MDT course for two types of leprosy: 6 months for PB and 12 months for MB both in baseline and end-line. On the other hand, the physicians and the other providers had inadequate knowledge on the duration of MDT at the baseline. The knowledge changed significantly though at the end-line. A higher proportion of physicians demonstrated increased knowledge (33% vs. 100%; *p* < 0.01) for PB cases and (42% vs. 100%; *p* < 0.01) for MB case respectively. For the other types of provider this change was (18% vs. 71%; *p* < 0.001) for PB cases and (13% vs. 57%; *p* < 0.001) for MB case respectively.

### Knowledge about drug use for adult PB case and MB case among service providers

The questions on individual doses and courses of the components of MDT for adult MB and PB cases were asked. The correct answer was Rifampicin 600 mg once a month, Clofazimine 300 mg once a month, and 50 mg daily and Dapsone 100 mg daily for the adult MB case and Rifampicin 600 mg once a month and Dapsone 100 mg daily for the adult PB case. Table 4 shows that TLCAs demonstrated significantly increased knowledge about doses and courses for the adult PB and MB cases at the end of the project compared to the beginning of the project (83% vs. 100%; *p* > 0.001). Similarly, physicians demonstrated significant increases in knowledge at the end of the project about drug dose for PB case (0% vs. 57%; *p* < 0.001) and MB case (0% vs. 43%; *p* < 0.01). Other service providers also demonstrated significantly increased knowledge about doses and courses for the adult PB at the end-line compared to the baseline (3% vs. 11%; *p* < 0.05). However, their change in knowledge was not significant about doses and courses for the adult MB cases at the end-line compared to the baseline (3% vs. 9%).

**Table 4:** Knowledge about drug use for adult PB case and MB case among service providers

	Physician			TLCA			Others*		
Knowledge about drug use for adult cases	Baseline (*n* = 12) % (90% CI)	End-line (*n* = 7) % (90% CI)	*P*-value	Baseline (*n* = 6) % (90% CI)	End-line (*n* = 7) % (90% CI)	*P*- value	Baseline (*n* = 80) % (90% CI)	End-line (*n* = 35) % (90% CI)	*P*-value
PB case	0%	57% (26.3–87.9)	***	83% (57.8–108.2)	100%	***	3% (−0.4–5.4)	11% (2.6–20.2)	*
MB case	0%	43% (12.1–73.7)	**	83% (57.8–108.2)	100%	***	3% (−0.4–5.4)	9% (0.8–16.4)	NS

*NS* Not significant

*p < 0.05; **p < 0.01; ***p < 0.001

Others* mean field workers and their supervisors

### Knowledge on types of leprosy services among service providers

Table 5 shows that TLCAs demonstrated significant increases in knowledge at the end-line compared to the baseline about supportive counseling (33% vs. 86%; *p* < 0.05) but not significantly increased about complication management (50% vs. 71%), whereas they had decreased knowledge regarding lab investigation and referral at the end-line. However, physicians had decreased knowledge regarding supportive counseling and lab investigation but slightly increased knowledge regarding referral and complication management at the end-line compared to the baseline. However, other service providers demonstrated decreases in the knowledge regarding supportive counseling, lab investigation and referral except complication management at the end-line.

**Table 5:** Knowledge on types of leprosy services among service providers

	Physician			TLCA			Others*		
Types of leprosy services	Baseline (*n* = 12) % (90% CI)	End-line (*n* = 7) % (90% CI)	*P*-value	Baseline (*n* = 6) % (90% CI)	End-line (*n* = 7) % (90% CI)	*P*-value	Baseline (*n* = 80) % (90% CI)	End-line (*n* = 35) % (90% CI)	*P*-value
Supportive counseling	92% (78.6–104.8)	29% (0.5–56.7)	***	33% (1.6–65.0)	86% (63.9–107.3)	*	64% (55.0–72.6)	46% (31.8–59.6)	*
Lab investigation	8% (−4.8–21.4)	0%	NS	33% (1.6–65.0)	14% (−7.5–36.1)	NS	5% (1.0–9.0)	0%	NS
Referral	25% (4.4–45.6)	29% (0.5–56.7)	NS	17%	0%	NS	60% (51.0–69.0)	29% (16.0–41.2)	***
Complication management	42% (18.3–65.1)	43% (12.1–73.7)	NS	50% (16.4–83.6)	71% (43.3–99.5)	NS	5% (1.0–9.0)	20% (8.9–31.1)	**

*NS* Not significant

*p < 0.05; **p < 0.01; ***p < 0.001

Others* mean field workers and their supervisors

## Discussion

The study findings demonstrated a significant increase of knowledge on types of leprosy, one cardinal sign, courses of MDT and drug use for the adult PB and MB cases and use MDT for leprosy treatment among primary and other service providers (community level service providers) at the end-line. This result indicates that the efforts of HSS project had a positive impact on knowledge about leprosy and its management among various types of service providers. Knowledge of service providers regarding types of leprosy, cardinal signs for diagnosis, treatment courses, and drug use for the diagnosed cases are essential to have an effective leprosy control program.

Knowledge regarding types and one cardinal sign mainly definite loss of sensation in a pale (hypopigmented) or reddish skin patch is important for diagnosis of leprosy. A study finding of Pakistan revealed that 76% of practitioners could recognize this sign of leprosy [4]. We found in our survey that 71% of physicians had knowledge about one cardinal sign for diagnosis of leprosy at the end-line. On the other hand, TLCAs had adequate knowledge about one cardinal sign for diagnosis of leprosy at the end-line (86%). The reason might be that TLCAs are front line healthcare providers who are traditionally engaged directly to detect leprosy case and to provide treatment and therefore probably more emphasis was given on their theoretical and practical training. Knowledge regarding course of MDT for MB and PB leprosy and drug use for the adult MB and PB cases is the most important and essential for treatment and control of leprosy. We found in our study that all TLCAs had adequate knowledge about course of MDT for MB and PB leprosy both in baseline and end-line. Because they are routinely engaged in day to day management and providing leprosy services in the country. A study in Pakistan, found that 45% of practitioners had knowledge regarding MDT [3]. Findings from other studies showed that physicians lacked awareness with the duration of leprosy treatment [5–8]. These findings are consistent with our end-line findings. Physicians did not demonstrate adequate knowledge about appropriate use for the adult MB and PB cases in both the baseline and the end-line. The reason might be that they are not directly involved for providing leprosy services. But according to the national guidance, physicians (Medical Officer-Disease Control) are responsible to confirm the diagnosis of leprosy by examining every suspect who had attended voluntarily to the MDT centre or referred by field workers and to initiate MDT and also to manage complicated cases [9].

Knowledge about leprosy services is essential for early diagnosis, treatment, and complication management which helps to prevent deformities and disabilities. Physicians and other service providers demonstrated increased knowledge about the use “MDT” for leprosy service at the end-line. The evaluation report of the HSS Project revealed that overall service providers had improved their knowledge and efficiency for leprosy services through the efforts of leprosy integration activities (Ahmed JU, Bangali AM: Evaluation report: 2015 and beyond: poverty reduction through strengthened health system 2013–2015, unpublished).

All service providers had trivial knowledge regarding lab investigation and referral at the end-line compared to the baseline. The reason might be that they did not maintain proper recording and reporting regarding referral and lab investigation at the baseline. Service providers referred the suspect cases verbally which was reported (higher proportion) by the respondents at the baseline. Referral slip was introduced during the leprosy integration activities under the HSS project which was used for referred suspect cases and counted as actual referral cases. This may be reflected at the end-line. Physicians and other service providers had insignificant knowledge regarding supportive counseling. The reason might be that they gave less emphasis on it during the leprosy integration activities and also they probably do not take part in any of the counseling activities. But supporting counseling is the most important activity for helping the leprosy patient for complete their MDT course and also help to prevent complication management/disabilities. TLCAs had adequate knowledge regarding supporting counseling at the beginning and at the end-line as they are the persons to engage full time in all spectrums of leprosy services starting suspecting cases, confirming diagnosis, starting MDT, follow up, and management of complication including counseling.

### Limitations of the study

Findings from this study should be viewed in light of several limitations. There was no control area and the present study adopted only pre-post evaluation approach. Data were obtained from only two districts out of 12 districts where the HSS programme was implemented. We used 90% CIs due to small sample. Further, the work was limited to providers with no input from program managers or individuals afflicted with leprosy.

## Conclusions

The leprosy integration activities under the HSS Project improved leprosy service and management related knowledge among providers included in the study. There was remarkable impact on the primary and community level service providers’ knowledge on types of leprosy, one cardinal sign, courses of MDT and drug use for the adult PB and MB cases and use MDT for leprosy treatment. Integration efforts should include on-job training for all providers during the implementation and phase out stages of integration to maintain the knowledge level, particularly during the current zero leprosy initiative and transition stage. Policy makers should follow-on with additional programs of integration that include providers, program managers, and patients to enhance leprosy service management and referral linkage in the high, medium and low leprosy burden areas. This study demonstrated that training improved knowledge among all cadres of service providers though varied among those who provided direct services and those who are engaged partially. Retention of knowledge in the long run was not measured and remained to be explored in future researches.

## Supplementary information

**Additional file 1.** Face Sheet for Service Provider Survey Questionnaire (DOCX 36 kb)

## Data Availability

There are restrictions on the availability of data sets due to agreements signed between Funding agency and icddr,b. However data are available from the authors upon reasonable request and with permission of icddr,b.

## Abbreviation

CIs: Confidence Intervals
DGHS: Directorate General of Health Services
FROs: Field Research Officers
G2D: Grade two Disabilities
HPSP: Health Population and Sector Programme
HSS: Health System Strengthening
icddr,b: international centre for diarrhoeal disease research, Bangladesh
MB: Multibacillary
MBDC: Mycobacterium Disease Control Programme
MDT: Multi-Drug Therapy
MoHFW: Ministry of Health and Family Welfare
NGOs: Non Government Organizations
NLP: National Leprosy Programme
NLTCC: National Leprosy and TB Coordinating Committee
NTCL: National Technical Committee for Leprosy
PABL: Person Affected By Leprosy
PB: Paucibacillary
TLCA: TB and Leprosy Control Assistant
WHO: World Health Organization
