# Supplementary material for: Knowledge on leprosy and its management among primary healthcare providers in two districts of Bangladesh
Source: BMC Health Serv Res. 2019 Nov 4;19:787. doi: 10.1186/s12913-019-4525-z (PMC6827226; doi:10.1186/s12913-019-4525-z)
Supplement: Supplementary file 1 — Additional file 1. Face Sheet for Service Provider Survey Questionnaire (DOCX 36 kb) [file 12913_2019_4525_MOESM1_ESM.docx]

**International Centre for Diarrheal Diseases Research, Bangladesh (icddr,b)**

**Situation analysis and strengthening integration of leprosy elimination activities into general health services in Bangladesh**

**Face Sheet for Service Provider Survey Questionnaire**

| **IDENTIFICATION** | | | | | | | | |
| --- | --- | --- | --- | --- | --- | --- | --- | --- |
| DIVISION  DISTRICT:  UPAZILA:  UNION/WARD  VILLAGE/MOHALLA/BLOCK  CLUSTER NUMBER  HOUSEHOLD NUMBER  RESPONDENT NUMBER  TYPE OF AREA: RURAL 1 URBAN 2 | | | | | | | |  |
| INTERVIEWER VISITS | | | | | | | | |
|  | 1 | | | 2 | 3 | | FINAL VISIT | |
| DATE |  | | |  |  | | Day Month Year | |
| INTERVIEWER’S NAME |  | | |  |  | | Interviewer's code | |
| RESULT CODE | COMPLETED =1  POSTPOND =2  NOT AT HOME =3  REFUSED =4  RESPONDENT INCAPACITATED =5  OTHERS =6 | | | | | | Result code | |
| NEXT VISIT:DATE |  | | |  |  | | Total no of visits | |
| TIME |  | | |  |  | |  |  |
| SUPERVISOR | | | FIELD EDITOR | | | | OFFICE EDITOR | |
| NAME | |  | NAME | | |  |  | |
| DATE | |  | DATE | | |  |  |  |

#

Time started for interview: Hour Minute

SECTION I: SOCIO-ECONOMIC CHARACTERISTICS

| **No.** | **QUESTIONS** | **CODING CATEGORIES** | **SKIP** |
| --- | --- | --- | --- |
| 101 | How old are you ? | Year |  |
| 102 | Sex | Male 01  Female 02 |  |
| 103 | Marital Status | Unmarried 01  Married 02  Widow 03  Divorce 04  Others (Specify) 05 |  |
| 104 | What is your religion ? | Islam 01  Hindu 02  Buddhist 03  Cristan 04  Others (Specify) 77 |  |
| 105 | What is your educational qualification ? (If illiterate then write 00) | Degree |  |

**SECTION II: KNOWLEDGE TOWARDS LEPROSY**

| **No.** | **QUESTIONS** | **CODING CATEGORIES** | **SKIP** |
| --- | --- | --- | --- |
| **Knowledge and conception of Leprosy** | | | |
| 201 | Would you please mention the types of leprosy? | Paucibacillary (PB) patients 01  Multibacillary (MB) patients 02 |  |
| 202 | What are the number of skin lesions for each type of leprosy | PB cases have up to five skin lesions 01  MB cases have six or more skin lesions 02 |  |
| 203. | What are the signs and symptoms of leprosy patient | Pale or reddish patches on the skin 01  Loss or decrease of feeling in the skin  patch 02  Numbness or tingling of the  hands or feet 03  Weakness of the hands, feet or eyelids 04  Painful or tender nerves 05  Swellings or lumps in the  face or earlobes 06  Painless wounds or burns on  the hands or feet 07  Others (Specify) 77  Don’t know 88 | 205 |
| 204 | Would you please mention at least one cardinal signs for diagnosis of leprosy | Definite loss of sensation in a pale  (hypopigmented) or reddish skin patch 01  A thickened or enlarged peripheral nerve 02  Loss of sensation 03  Presence of acid-fast bacilli  in skin smear 04  Others (Specify) 77 |  |
| 205. | What type of complication may arise from leprosy? | Wounds or ulcers on the hands or feet 01  Marked redness of the eye 02  Incomplete eye closure 03  A claw hand 04  A drop foot 05  Loss of tissue, such as fingers or  toes shortened or missing 06  Others (Specify) 77 |  |
| 206. | What types of services should be provided to a person affected with leprosy? | MDT 01  Supportive counseling 02  Lab investigation 03  Referral 04  Complication management 05  Others (Specify) 77 |  |
| 207. | **I would request you to answer the following questions: please tell whether it is true or false.** | |  |
|  | a. Leprosy is not hereditary | True 01  False 02  Don’t know 88 |  |
|  | b. It is completely curable | True 01  False 02  Don’t know 88 |  |
|  | c. If untreated it leads to deformity | True 01  False 02  Don’t know 88 |  |
|  | d. There is a vaccine that can prevent a person from getting leprosy | True 01  False 02  Don’t know 88 |  |
|  | e. Leprosy patient should lead a normal life. | True 01  False 02  Don’t know 88 |  |
|  | f. Leprosy is no longer infectious once treatment has begun | True 01  False 02  Don’t know 88 |  |
|  | g. Skin patches take time to disappear. | True 01  False 02  Don’t know 88 |  |
|  | h. Existing disability may or may not improve with treatment. | True 01  False 02  Don’t know 88 |  |
|  | i. Recurrent of disease is rare | True 01  False 02  Don’t know 88 |  |
| **III. Skills for service delivery** | | | |
| 301 | Have you receive any training on leprosy | Yes 01  No 02 | 305 |
| 302. | If yes, from where did you receive training | NLEP 01  Lepra 02  NGO 03  Abroad 04  Others 77 |  |
| 303 | Do you think the training was adequate | Yes 01  No 02 | 401 |
| 304. | Do you think you need further training on leprosy | Yes 01  No 02 |  |
| 305. | Would you please mention about the course of leprosy treatment | MDT for 6 months for PB 01  MDT for 12 months for MB 02  Common side-effects include red  urine and darkening skin 03  Tablets must be taken every  day at home 04  New blister-pack is needed  every 28-days 05 |  |
| 306 | What are the drugs you use in MDT for adults MB case | Rifampicin: 600 mg once a month 01  Clofazimine: 300 mg once  a month, and 50 mg daily 02  Dapsone: 100 mg daily 03 |  |
| 307 | What are the drugs you use in MDT for adults PB case | Rifampicin: 600 mg once a month 01  Dapsone: 100 mg daily 02 |  |
| 308 | What are the drugs you use in MDT for child (ages 10 – 14) MB leprosy | Rifampicin: 450 mg once a month 01  Clofazimine: 150 mg once a month,  and 50 mg every other day 02  Dapsone: 50 mg daily 03 |  |
| 309 | How do you treat a relapse case of leprosy | Same treatment as new cases 01  Others 77 |  |
| 310 | How do you treat a default case of leprosy | Same treatment as new cases 01  Others 77 |  |
| 311 | How do you treat a change in classification from PB to MB case of leprosy | Full course of MB treatment 01  Others 77 |  |
| 312 | What points you address during counseling of a leprosy patient | Consultations and treatment are  free-of-charge 01  Frequency of leprosy patient should  attend the clinic 02  Leprosy is no longer infectious once  treatment has started 03  Close contacts may develop leprosy 04  Skin patches take time to disappear 05  Patches can suddenly become red  and swollen again 06  There may be pain or numbness in  the limbs 07  There may be weakness of  hand or feet 08  There may loss of vision, pain  or redness 09  New disability can occur at any  time but it can be treated 10  Existing disability may or may  not improve with treatment 11 |  |
| 313 | Do you follow the standard guideline while treating a leprosy patient | Yes 01  No 02 |  |
| 314 | If no, please mention the reasons | Standard guideline is not available 01  Lack of trained staff member 02  Lack of specialized providers 03  Lack of laboratory facilities 04  Lack of diagnostic instruments 05  Inadequate amounts of MDT  drugs MDT drugs are not free of cost 06  IEC materials are not available 07  Treatment register not available 08  Referral linkage not strong 09  Others 77 |  |
| 315 | How do you assess the disability from leprosy | Grade 0: no disability 01  Grade 1 : loss of sensation in  the hand or foot 02  Grade 2: visible damage  in eyes, hand, foot 03 |  |
| 316 | When do you refer a case of leprosy routinely | Leprosy is suspected but the  diagnosis is uncertain 01  Suspected relapse 02  Long-standing disability  which requires surgery 03  Long-standing disability which  requires rehabilitation intervention 04  Others 77 |  |
| 317 | Where do you refer these cases | District hospital 01  Medical College hospital 02  Private clinic/doctor 03  Others (Specify) 77 |  |
| 318 | When do you refer a case of leprosy on emergency basis | Severe reversal reactions 01  Reversal reactions overlying  a major nerve trunk 02  Neuritis 03  Erythema Nodosum Leprosum (ENL)  reactions 04  Severe infection of the hand or foot 05  Recent loss of visual acuity 06  A painful red eye 07  Recent inability to close the eye 08  Others 77 |  |
| 319 | Where do you refer these cases | District hospital 01  Medical College hospital 02  Private clinic/doctor 03  Others (Specify) 77 |  |

| **IV. Attitude towards leprosy person** | | | | | |  |
| --- | --- | --- | --- | --- | --- | --- |
| 401. | In your opinion what are the rights do leprosy person should have? | | Access the information they need 01  Safe home living conditions 02  Go to school / study/work 03  Access health care 04  Access assistive device 05  Relationships with others 06  Make decisions about their own life 07  Opinion counts in family discussions 08  Opinion counts in community  discussions 09  Be treated the same way as anyone else...10  Get married if they want to 11  Have children if they want to 12  Other (Please specify) 77  Do not know 88 |  | | |
| **I would like to request you to answer the following questions, please tell me whether you agree or disagree**  **disagree** | | | | | | |
| 402. | Person with leprosy should have different room for service | | Agree 01  Disagree 02  Don’t know 88 |  | | |
| 403. | I am afraid of getting leprosy while treating person with leprosy | | Agree 01  Disagree 02  Don’t know 88 |  | | |
| 404. | Person with leprosy should not be excluded from social gathering | | Agree 01  Disagree 02  Don’t know 88 |  | | |
| 405. | Person with leprosy should have the same respect or standing as others in the  community | | Agree 01  Disagree 02  Don’t know 88 |  | | |
| **V. Exposure to outreach activités** | | | | | | |
| 501. | Do you know about any outreach activities for the leprosy patient? | | Yes 01  No 02 | 506 | | |
| 502. | Are you a member of a leprosy group? | | Yes 01  No 02 |  | | |
| 503. | Do you think the outreach activities useful? | | Yes 01  No 02 | 505 | | |
| 504. | If yes, why? | | Reduce stigma 01  Increase knowledge  about disease 02  Increase knowledge about  health facility 03  May cause early treatment 04  Reduce disability 05  Don’t No 06 |  | | |
| 505. | If no, why? | | No clear message 01  Target of audience is poor 02  Not organized 03  Inadequate contact 04  Don’t know 88 |  | | |
| 506 | | **INTERVIEWER: Before leaving the respondent check the questionnaire carefully. Finish the interview by giving thanks.** | | |  | |

Time finished Hour Minute
